# Staying or Leaving the Front Line: Identity, Support, and Meaning among Child Protection Professionals in Hungary

**DOI:** 10.1192/j.eurpsy.2026.10772

**Published:** 2026-07-31

**Authors:** A. Kun, J. Páskuné Kiss

**Affiliations:** 1 Doctoral School of Human Sciences; 2 Department of Counselling, Developmental and School Psychology, University of Debrecen, Debrecen, Hungary

## Abstract

**Introduction:**

Workforce instability in child protection threatens service quality and staff mental health (burnout, moral distress). Profile-based evidence is scarce in Central and Eastern Europe, despite international concern about retention (Burns & Christie Child Youth Serv Rev 2013; 34:1115–22; Cook et al. CRCF Briefing 2022; European Social Network. Protecting Children. 2024).

**Objectives:**

To identify psychosocial profiles of child protection professionals based on identity, support, and meaning, and to examine their relation to exit risk, enriched by qualitative narratives.

**Methods:**

Survey of Hungarian residential child protection staff (N = 90). Measures: MGIS – solidarity subscale (used exploratorily in Hungary; α = 0.98), MSPSS (α = 0.85), and PERMA-Profiler subscales ‘meaning’ and ‘relationships’ (α = 0.89). Exit intention was assessed with items on career plans and active job search. Analyses included descriptive statistics, Mann–Whitney U, Spearman correlations, and Ward + k-means cluster analysis. Additionally, 15 semi-structured interviews were analysed thematically (Braun & Clarke. Qual Res Psychol 2006;3:77–101).

**Results:**

Three clusters emerged:

1) Embedded but strained (n = 56; 62.2%) – strong solidarity and meaning, but only moderate external support.

2) Isolated strugglers (n = 29; 32.2%) – lowest support and meaning, identity under strain, highest exit risk.

3) Supported outsiders (n = 5; 5.6%) – weak professional identity and meaning, but strong family/friend support.

Solidarity was significantly lower among those at exit risk (U = 742.5, p < 0.001). Interviews illustrated these profiles: “Nobody listens to us, and nobody cares” (struggler); “My colleagues keep me in the profession; without them I would have quit” (embedded); “I don’t stay because of the workplace, I stay because of my family” (outsider).

**Conclusions:**

Exit risk reflects identity–support–meaning profiles rather than stress alone. These findings align with European evidence on workforce instability and highlight intervention points beyond individual stress management. Strategies that strengthen solidarity, reinforce work meaning, and leverage external support may prevent burnout and turnover, stabilising child-facing mental-health services.

**Disclosure of Interest:**

None Declared

